# An Analytic Model for the Success Rate of a Robotic Actuator System in Hitting Random Targets

**DOI:** 10.3390/s151129346

**Published:** 2015-11-20

**Authors:** Stuart Bradley

**Affiliations:** Physics Department, University of Auckland, Private Bag 92019, Auckland 1010, New Zealand; E-Mail: s.bradley@auckland.ac.nz; Tel.: +64-9373-7599 (ext. 88886)

**Keywords:** robotic platform, robotic actuator, robotic targeting, random targets

## Abstract

Autonomous robotic systems are increasingly being used in a wide range of applications such as precision agriculture, medicine, and the military. These systems have common features which often includes an action by an “actuator” interacting with a target. While simulations and measurements exist for the success rate of hitting targets by some systems, there is a dearth of analytic models which can give insight into, and guidance on optimization, of new robotic systems. The present paper develops a simple model for estimation of the success rate for hitting random targets from a moving platform. The model has two main dimensionless parameters: the ratio of actuator spacing to target diameter; and the ratio of platform distance moved (between actuator “firings”) to the target diameter. It is found that regions of parameter space having specified high success are described by simple equations, providing guidance on design. The role of a “cost function” is introduced which, when minimized, provides optimization of design, operating, and risk mitigation costs.

## 1. Introduction

Autonomous robotic systems are increasingly being used in a wide range of applications such as precision agriculture, medicine, and the military. These systems frequently include tracking, scheduling, and target recognition capabilities, and actuators which interact with the target in some way. In the current paper, this interaction will be called “hitting the target”, and the paper is concerned with the success rate of hitting randomly distributed targets.

For example, a survey of the state of the art in unmanned service units in agricultural environments [1] presented the four core abilities of such vehicles when performing agricultural tasks: detection, guidance, mapping, and action. In precision agriculture, there is increasing interest in optimization of, for example, pest control sprays [2]. A good discussion is given by [3] of the management of robotic agricultural systems. They consider the use of “implements” in a generic sense where, for example, typical implements are a sprayer boom, a mechanical-thermal tool and an air-blast sprayer, which here will be called actuators. Reference [4] considered the statistical nature of autonomous platform control and tracking, but not the statistical nature of the random presentation of targets (weeds). Sophistication is increasing in precision agriculture, and the design, development and testing of a new generation of automatic and robotic systems for both chemical and physical pest management [5] would benefit from having a model of target hit success rate, the subject of the present paper.

There are an increasing number of high-quality studies obtaining measurements of the effectiveness of automated agricultural systems, for example to reduce chemical loss and cost by applying the right amount of spray at the right time and location [6]. Some aspects of navigation and feature recognition for autonomous orchard robots are very well developed, both in simulations and theoretically [7]. The state of the art in robotic viticulture is described by [8], including aspects of pruning and spraying. This includes success in hitting the target but, in common with virtually all the available literature, this aspect is not covered in any depth. Large savings, such as 15% for an olive grove spray application [9], can be achieved by optimizing spray platform motion in relation to circular target diameter and spacing. The success rate for hitting regularly spaced circular targets has been discussed by [10] as a function of winds affecting spray drift, which has some overlap with the problem of a moving robotic platform, but does not address the success rate in hitting random targets.

Within medical science there is considerable interest in optimizing targeting of, for example, tumors [11]. The probability of successfully hitting a target in 3D space has been considered as a general problem by [12], who included aspects of the Poisson probability analysis we develop later. However, relative motion of platform and targets is not included.

Very detailed formal analyses exist, such as [13], for the problem of *finding* a target, but this is not the same problem as that of *hitting* a target which randomly presents itself. Target tracking is also a different problem [14].

Across the multiplicity of applications, there is considerable commonality, including the absence of analytic models for the success rate of hitting targets. Such models, if available, can be useful tools guiding improved system development. Such improved design is ultimately about reducing costs (or improving efficiency). Task scheduling analyses [15,16] aim to reduce the “cost”, known as Makespan, Tardiness, or Flowtime, which can be a complex measure of system resource use and penalties. As part of the model presented in the current paper, the nature of a “cost function” which ideally should be minimized, is also considered.

## 2. Geometry

**Figure 1:**
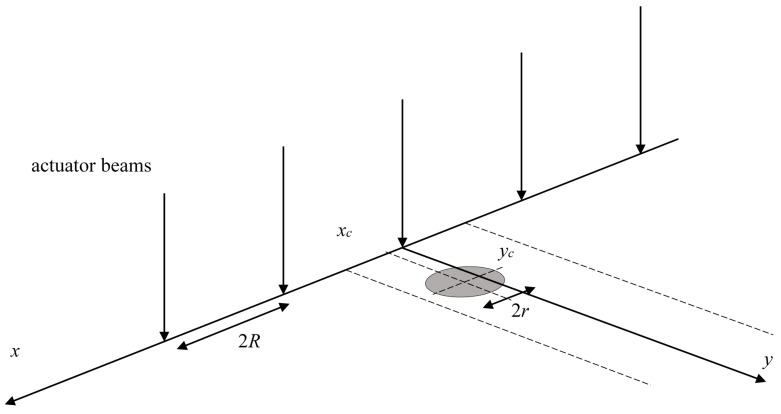
The geometry of evenly-spaced actuator beams and circular targets.

Circular targets of radius *r* are uniformly distributed on the *x*-*y* plane. Actuator “beams” (or ability to hit a target) point in the negative *z* direction, so that their beams pass through (*x*, *y*) = …, (−4*R*, *vt*), (−2*R*, *vt*), (0, *vt*), (2*R*, *vt*), (4*R*, *vt*), … where the line of actuators is moving transverse to the line at speed *v* in the *y* direction, and *t* is time (see Figure 1).

**Figure 2:**
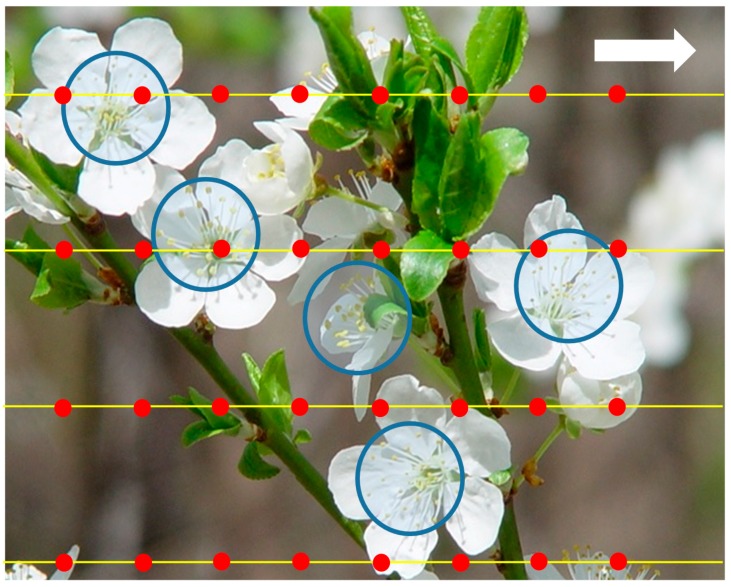
An illustration of the targeting problem, based on spray pollination of apple blossoms. The target areas are shown as blue circles, the actuator paths are shown as yellow lines, and the positions hit by the spray are shown as red dots.

Instantaneously, any targets whose centers lie within a radius of *r* from an actuator will be hit. If the actuator beam has a finite radius, this can be accounted for by making *r* the sum of actuator and target radii. Assume the actuator can be commanded to “fire” at times *t* = 0, τ, 2τ, … In time τ a swath 2*R* wide and *v*τ long is swept per actuator. Figure 2 shows a hypothetical example of robotic pollination of apple blossoms using spray actuators. The large white arrow indicates the direction of motion of the spray boom and the yellow lines show the paths of four sprayers. These spray at regular intervals, shown by the red dots. The target circle is not the full blossom, but rather a circle encompassing the stamens, shown as a blue circle. Five targets are shown, ignoring those blossoms not fully open or obscured behind leaves. It can be seen that in this example only three of the five targets are hit.

The problem can be handled using scaled variables, so that it is easier to see what relationships between geometric parameters are important, and so that the results can more easily be applied to the wide range of potential applications. There are two dimensionless scales of significance. The first is the ratio ρ of actuator spacing, 2*R*, to target diameter, 2*r*. The second, γ, is a measure of how far the actuator platform moves in the time between fire commands, compared with the target diameter:

(1) $$\rho =\frac{2R}{2r},\;\;\gamma =\frac{v\tau }{2r}$$

**Figure 3:**
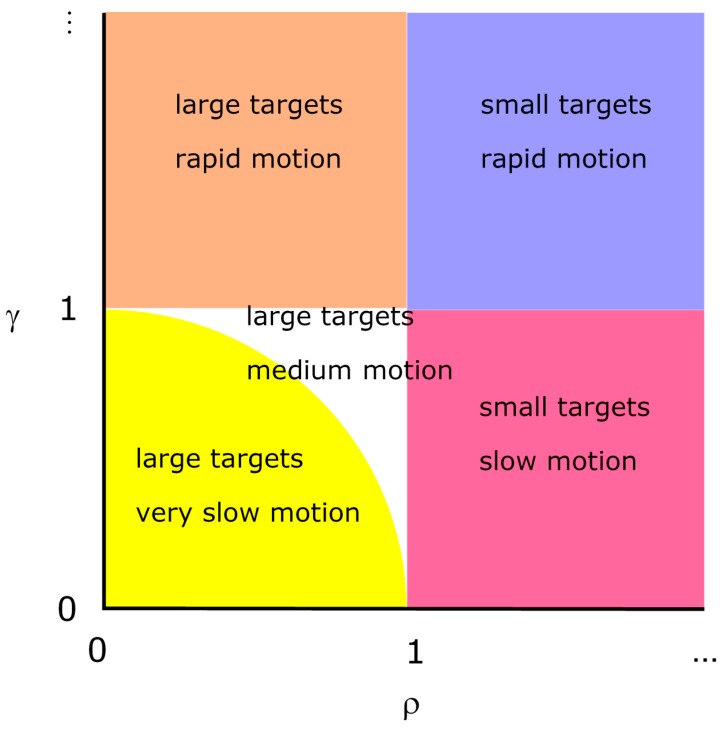
The five regions of parameter space, ρ, γ which define target success.

The unit of length can be considered as being 2*r*, the target diameter. There are four distinct regions of parameter space, as shown in Figure 3 and discussed in the following sub-sections.

### 2.1. Small Targets and Slow Motion

**Figure 4:**
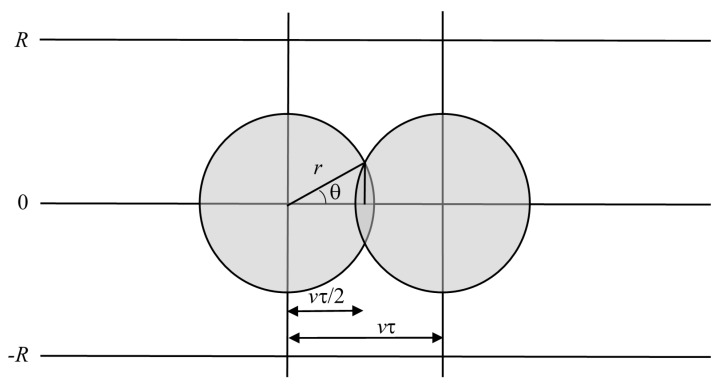
The circular areas within which target centers lie for successful hits, for two successive actuator firings.

The possible area for targets hit at two successive actuator firings is shown in Figure 4.

**Figure 5:**
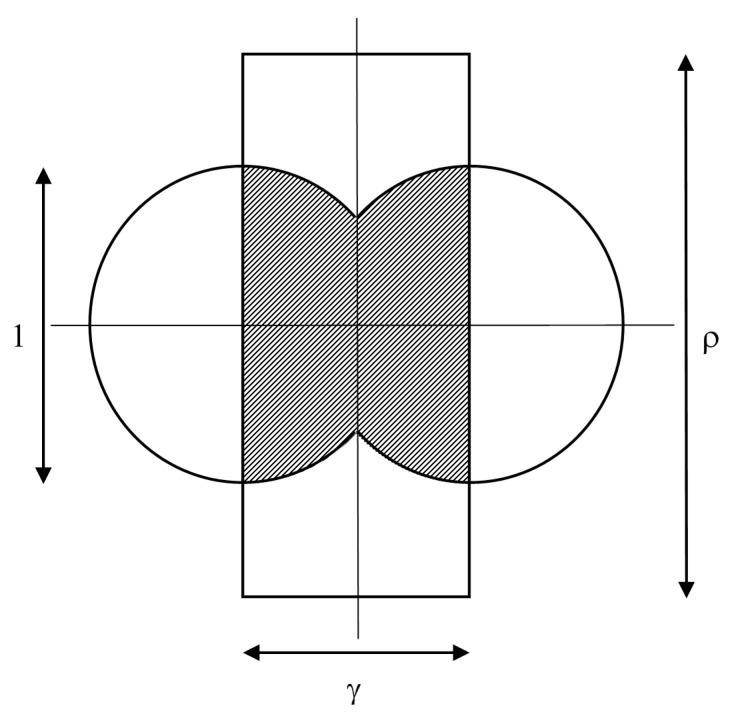
The shaded area shows the region in which a target center can lie for the target to be successfully hit, whereas the rectangular area is the swath swept out by an actuator, travelling left to right, during the time between fire commands.

The swept area is 2*Rv*τ or, in dimensionless terms, ργ. For this case, 1 ≤ *ρ*, *γ* ≤ 1, and the area between two firings which gives successful hits is the combination of the two semicircles between the two firings shown in Figure 4, but without the overlap of area. This is 4 × [(area of sector of angle π/2 − θ) + area of triangle shown in Figure 4)], or:

(2a) $$4\left[\left(\frac{\pi /2-\theta }{2\pi }\right)\pi r^{2}+\frac{1}{2}\frac{v\tau }{2}\sqrt{r^{2}-{\left(\frac{v\tau }{2}\right)}^{2}}\right]=\pi r^{2}\left[1-f\left(\gamma \right)\right]$$

where:

(2b) $$f\left(\gamma \right)=\frac{2}{\pi }\left({\text{cos}}^{-1}\gamma -\gamma \sqrt{1-\gamma^{2}}\right)$$

and cos(θ) = (*v*τ/2)/*r* = γ. The dimensionless version is shown in Figure 5. Dividing by the area swept out gives a fractional success rate of hitting a target of:

(3) $$h=\frac{\pi }{4\rho \gamma }\left[1-f\left(\gamma \right)\right]$$

### 2.2. Small Targets and Fast Motion

**Figure 6:**
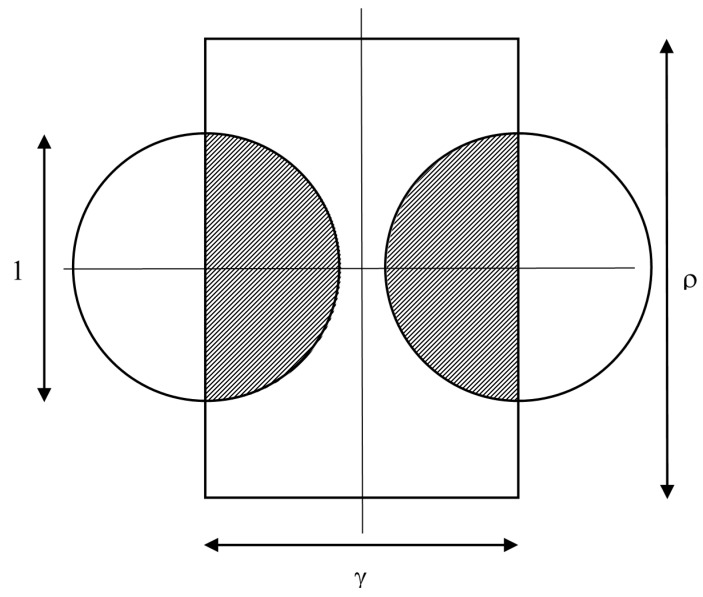
As for Figure 5, but with non-overlapping regions for successful hits.

For this case, 1 ≤ *ρ*, 1 ≤ *γ*, the dimensionless area of each half of the shaded region in Figure 6 is simply that of a semicircle, π/4. The fractional success rate of hitting a target is:

(4) $$h=\frac{\pi }{4\rho \gamma }$$

### 2.3. Large Targets and Very Slow Motion

**Figure 7:**
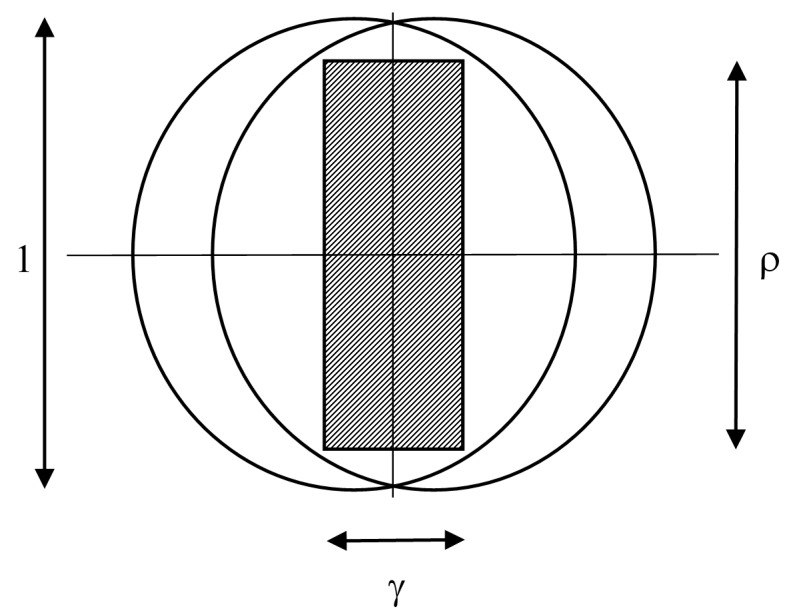
As for Figure 5, but with the swept area small compared to target size.

As shown in Figure 7, this is the case of actuators closely spaced, in comparison with the target size, and motion so slow that all targets are hit, and $\rho \leq 1,\;\gamma \leq \sqrt{1-\rho^{2}}$:

(5) h=1

### 2.4. Large Targets and Medium Speed Motion

**Figure 8:**
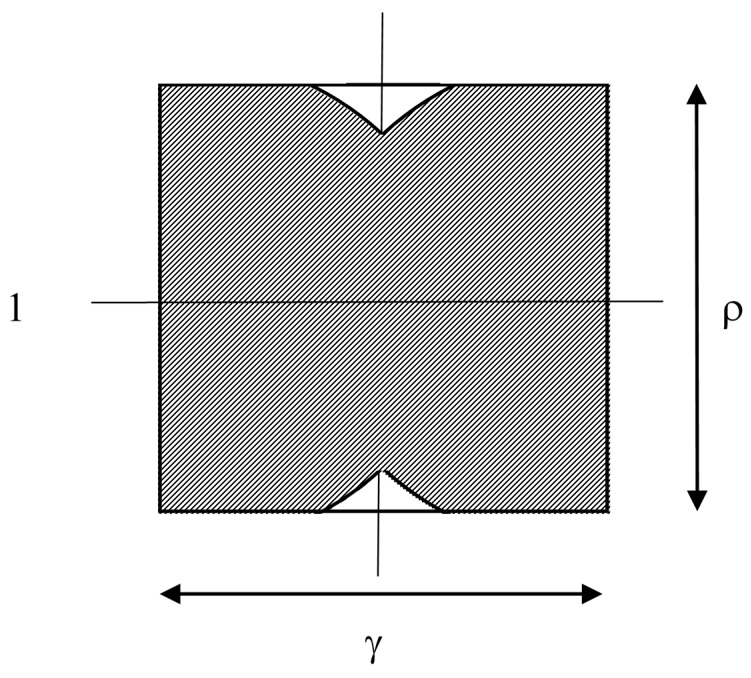
As for Figure 5, but with closer actuator spacing.

When $\rho \leq 1,\;\sqrt{1-\rho^{2}}\leq \gamma \leq 1$, shown in Figure 8, the shaded area is similar to the 1 ≤ *ρ*, *γ* ≤ 1 case described in Section 2.1, but cap areas are also removed from the top and bottom of the semicircles. These cap areas are defined by ρ instead of γ, but the geometry is similar, giving as a result:

(6) $$h=\frac{\pi }{4\rho \gamma }\left[1-f\left(\gamma \right)-f\left(\rho \right)\right]$$

Therefore the same function, *f*, can be used for truncating the semicircles in each direction.

### 2.5. Large Targets and Rapid Motion

**Figure 9:**
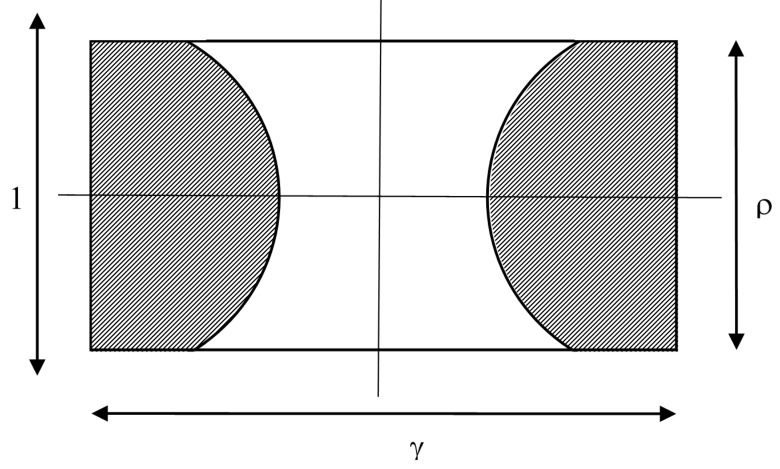
As for Figure 5, but with closer actuator spacing.

In the case of *ρ* ≤ 1, 1 ≤ *γ* the semi-circular areas are only reduced at the top and bottom in Figure 9, giving:

(7) $$h=\frac{\pi }{4\rho \gamma }\left[1-f\left(\rho \right)\right]$$

### 2.6. Discussion of Combined Performance

Combining the results from the four regions of parameter space gives, for the success rate *h* of hits on targets:

(8) $$h=\begin{array}{ccc} \frac{\pi }{4\rho \gamma }\left[1-f\left(\gamma \right)\right] & & 1\leq \rho ,\gamma \leq 1\\ \frac{\pi }{4\rho \gamma } & & 1\leq \rho ,\gamma \geq 1\\ 1 & & \rho \leq 1,\gamma \leq \sqrt{1-\rho^{2}}\\ \frac{\pi }{4\rho \gamma }\left[1-f\left(\gamma \right)-f\left(\rho \right)\right] & & \rho \leq 1,\sqrt{1-\rho^{2}}\leq \gamma \leq 1\\ \frac{\pi }{4\rho \gamma }\left[1-f\left(\rho \right)\right] & & \rho \leq 1,\gamma \geq 1 \end{array}$$

**Figure 10:**
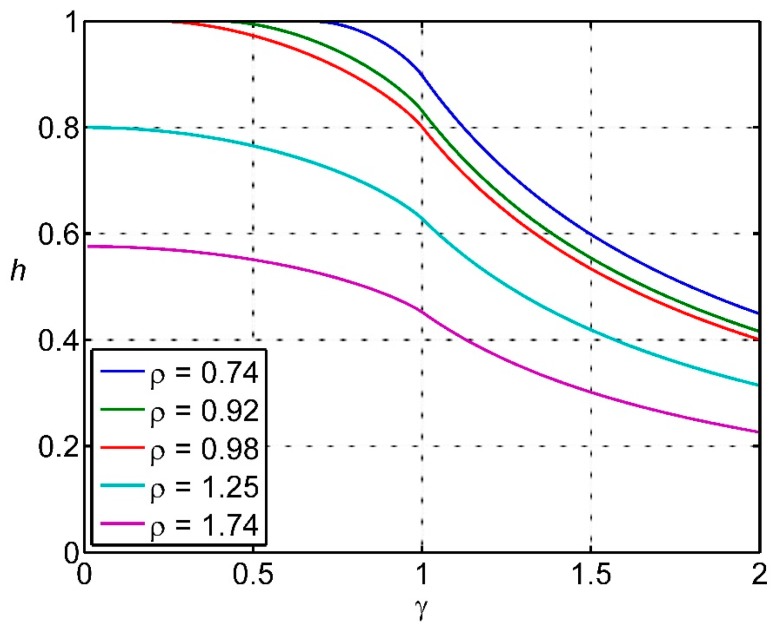
The fractional success rate *h* for hitting targets under different conditions of target size and platform speed.

This equation is plotted in Figure 10. For the actuator spacing less than a target diameter, 100% hit rate is achieved providing γ^2^ ≤ 1 − ρ^2^, or *v*τ ≤ [(2*r*)^2^ − *R*^2^]^1/2^. Since speed of completing a task is generally a factor, this means that the actuator platform speed should be set according to *v*τ = [(2*r*)^2^ − *R*^2^]^1/2^ because there is no penalty in doing so.

**Figure 11:**
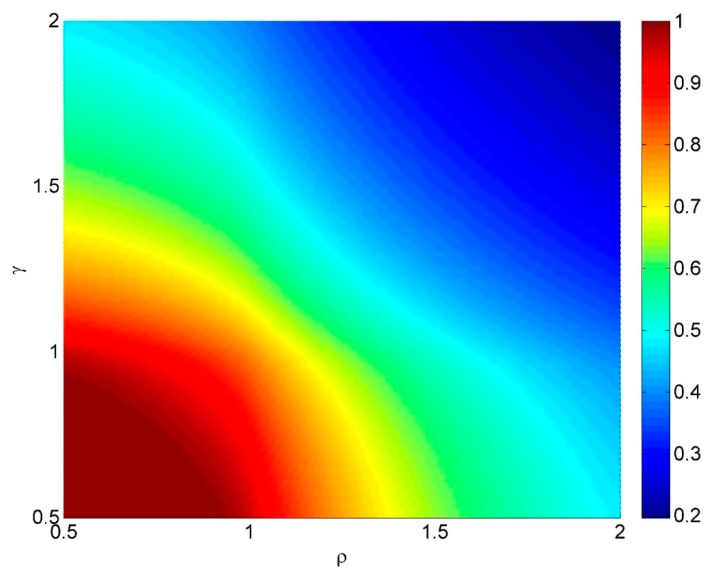
The dependence of fractional success rate *h* on the actuator spacing parameter ρ and the platform speed parameter γ. Colors range from blue for *h* = 0.2 to brown for *h* = 1.

If the actuator spacing is greater than a target diameter, then the maximum hit success rate is achieved at the lowest *v*τ and has a value of *h* = 1/ρ = target diameter/actuator spacing. This can be used as a very simple metric for design. The dependence of fractional success rate *h* on the actuator spacing parameter ρ and the platform speed parameter γ is shown in Figure 11.

## 3. The Cost Function

The least sophisticated design criterion might be that the success rate for hitting targets, *h*, should be greater than some lower limit *h*_0_. Such an approach ignores detailed considerations of real operating costs, and is probably justified on the basis that “it feels OK”. For high success rates *h*, Figure 10 and Figure 11 show that γ is most likely less than 1. From Equation (8) there is a region of parameter space (ρ, γ) which satisfies each *h* ≥ *h*_0_. The problem can be simplified by approximating [1 − *f*(γ)]/γ by a quadratic:

(9) $$\begin{array}{ll} \rho h_{0} & \leq \frac{\pi }{4\gamma }\left[1-f\left(\gamma_{0}\right)\right]\\ & \leq 1+\frac{1}{10}\left(\frac{\gamma_{0}}{2}\right)-{\left(\frac{\gamma_{0}}{2}\right)}^{2} \end{array}$$

where γ_0_ is the upper limit of γ which will give a hit rate above *h*_0_ if 1 ≤ ρ. Solving the quadratic shows that, for this case:

(10) $$1\leq \rho \leq \frac{1}{h_{0}}$$

imposes a range of acceptable values for the ratio of actuator spacing to target diameter. For example, if it is decided that the hit success rate should be above 95%, then:

$$1\leq \frac{\text{actuator}\;\text{spacing}}{\text{target}\;\text{diameter}}\leq 1.05$$

which is a tight design specification on actuator spacing. The design specification on spacing becomes tighter for higher minimum success rates *h*_0_.

**Figure 12:**
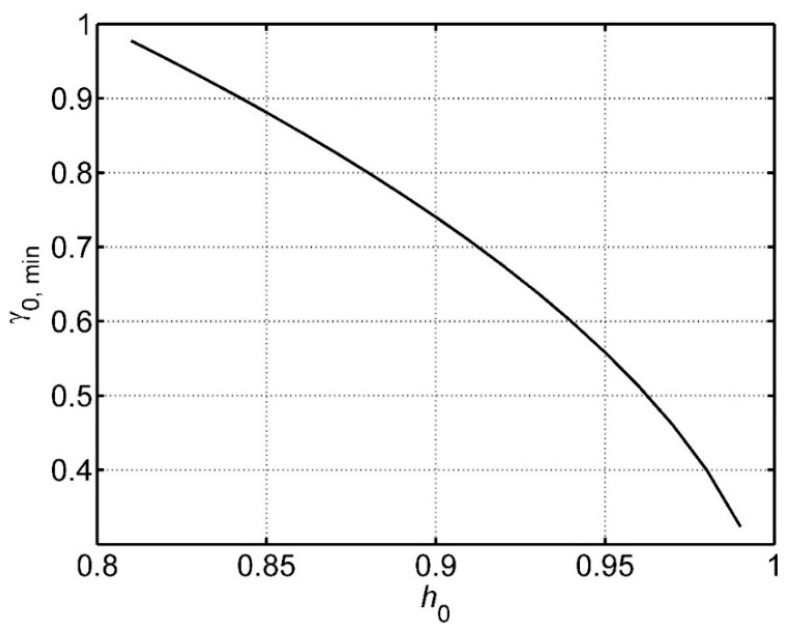
The lowest value γ_0,min_ for which a given hit success rate *h*_0_ can be achieved, when the actuator spacing is less than the target diameter.

For the case $\rho \leq 1,\;\sqrt{1-\rho^{2}}\leq \gamma \leq 1$ a cubic results in:

(11) $${\left(\frac{\gamma_{0}}{2}\right)}^{3}-\frac{1}{10}{\left(\frac{\gamma_{0}}{2}\right)}^{2}+\left(\rho h_{0}-1\right)\left(\frac{\gamma_{0}}{2}\right)+\frac{\pi }{8}f\left(\rho \right)=0$$

but only one of the three distinct real solutions is valid. No solution exists for *h*_0_ < *h*_min_ = 4/5. Also, for each *h*_0_ > 4/5, there is a limited range of the minimum γ, as shown in Figure 12.

This region of platform speed for which a given hit rate is possible can be approximated by:

(12) $$1-\frac{5}{2}\left(h_{0}-h_{\text{min}}\right)\leq \gamma_{0}\leq 1$$

For the simple design criterion of a minimum acceptable success rate, there is therefore an upper limit on actuator spacing, if the spacing is greater than the target diameter, and a lower limit on platform speed if the actuator spacing is less than the target diameter.

Clearly high success in targeting occurs at low speed, high command rate, and close spacing of actuators, but the design process is one of balancing the penalty of lower hit rate against the penalty of operating at lower speed (*i.e.*, taking longer over a task), and the higher component count of more actuators. This means that, in practice, a “cost function”, *c*(*h*, ρ, γ), needs to also be included with a diagram such as Figure 10 since otherwise it might be considered that the optimum design is very low ρ and very low γ.

As an illustration, which is not necessarily realistic, it might be that the cost of missing targets is proportional to 1 − *h*, the cost of closer spacing of actuators is proportional to 1/ρ, and the cost of taking longer to complete the task is proportional to 1/γ. Then the cost function would have the form:

(13) $$c\left(h,\rho ,\gamma \right)=\frac{1-h}{{\left(\rho \gamma \right)}^{u}}$$

where the power *u* has been included to allow for expensive spray material, for example (the least sophisticated design criterion corresponds to *u* = 0).

**Figure 13:**
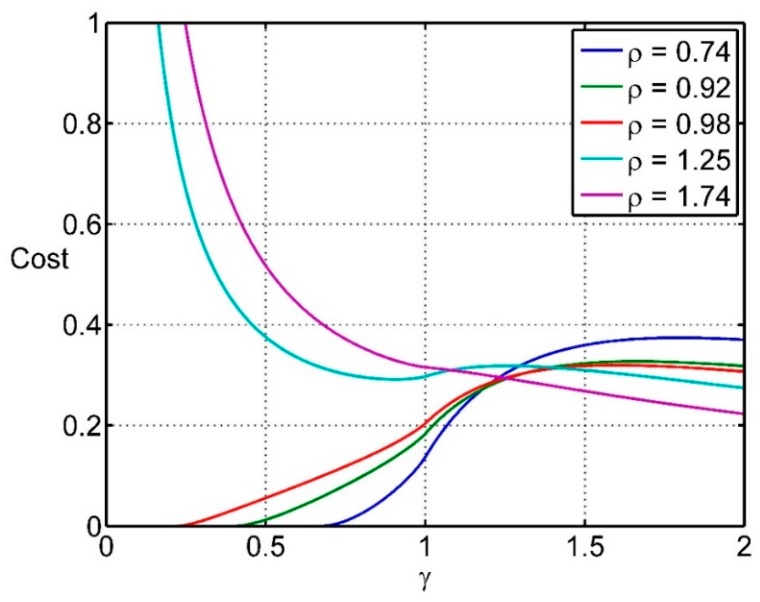
An illustration of a simple cost function applied to the targeting problem.

**Figure 14:**
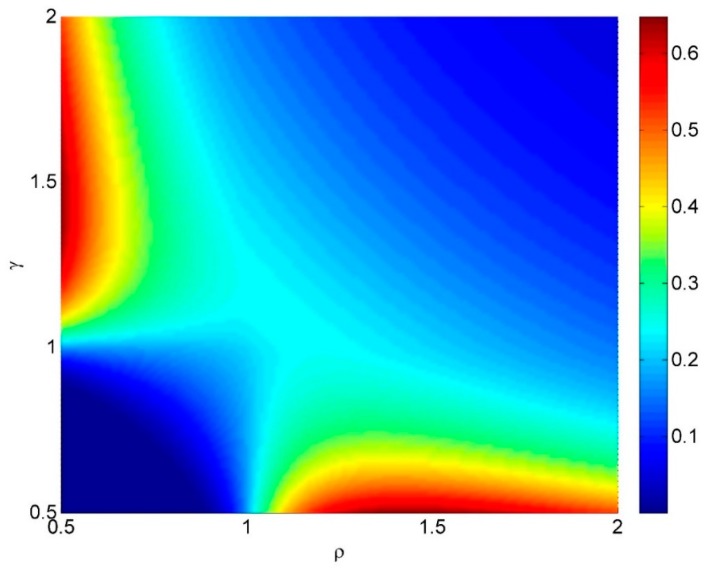
An illustration of a simple cost function applied to the targeting problem.

This simple cost function with *u* = 1 is plotted in Figure 13 and Figure 14. For this cost function the cost is low if the distance travelled between fire commands is much less than the target spacing, *and* the actuator spacing is less than the target diameter. There is little difference in hit rate *h* or in cost between ρ = 0.74 and ρ = 0.98, so providing the actuator spacing is less than the target diameter, reasonable performance can be obtained.

An example of the design considerations is the WEEDit agricultural spraying system [17]. This system comprises fast solenoid actuated weed sprayers spaced 1 m apart on a 36 m horizontal boom. The physical parameters are *R* = 0.5 m, *r* = 0.5 m, *v* = 6 m/s, and τ = 0.006 s. From Equation (6), with ρ = 1 and γ = 0.036, the predicted hit success is an extremely high *h* = 0.9998. However, such fast acting solenoids are very expensive, and the same hit rate *h* could be achieved, for example, with marginally closer spacing of sprayers with ρ = 0.90 and much slower acting solenoid valves γ = 0.43. Note that the actual cost function for such systems is commercially sensitive.

## 4. Probability of Success

Equation (8) gives the *mean* success rate for hitting targets under the full range of actuator spacing, target size, platform speed, and firing rate. But this equation does not give the probability of getting, for example, a very bad result in a particular run. To estimate such statistics requires a model of the probability *p*(*n*) of hitting *n* targets within an area. This estimation requires another parameter, the mean number λ of targets whose centers lie within a unit area. This problem satisfies the criteria for a Poisson probability distribution, where:

(14) $$p\left(n\right)=\frac{\lambda^{n}e^{-\lambda }}{n!}$$

In Section 2 the non-dimensional swept area is *S* = ργ and the hatched area is $\bar{h}S$, where $\bar{h}$ is the mean hit rate given by Equation (8). The probability of *m* targets in the “missed” area $\left(1-\bar{h}\right)S$ is:

(15a) $$p_{M}\left(m\right)=\frac{{\left[\lambda S\left(1-\bar{h}\right)\right]}^{m}e^{-\lambda S\left(1-\bar{h}\right)}}{m!}$$

and the probability of *n* targets in the “hit” area $\bar{h}S$ is:

(15b) $$p_{H}\left(n\right)=\frac{{\left(\lambda \bar{h}S\right)}^{n}e^{-\lambda \bar{h}S}}{n!}$$

Variables *m* and *n* are independent. The success ratio for hits is a random variable:

(16a) $$h=\frac{n}{m+n}$$

or:

(16b) $$m=\left(\frac{1-h}{h}\right)n$$

The probability density function of *h* is:

(17) $$g\left(h\right)=\sum_{n=0}^{\infty }p_{M}\left(\frac{1-h}{h}n\right)p_{H}\left(n\right)$$

**Figure 15:**
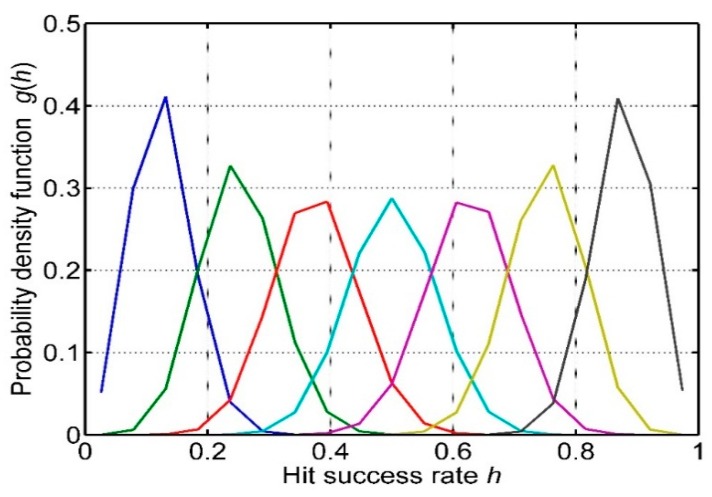
The probability density function for hit success rate *h* from Equation (12) for seven values of $\bar{h}$.

As an example, assume λ = 50 target centers per unit area and a swept area *S* = 1 m^2^. The pdf of hit success rate *h* is shown in Figure 15 for several values of $\bar{h}$.

**Figure 16:**
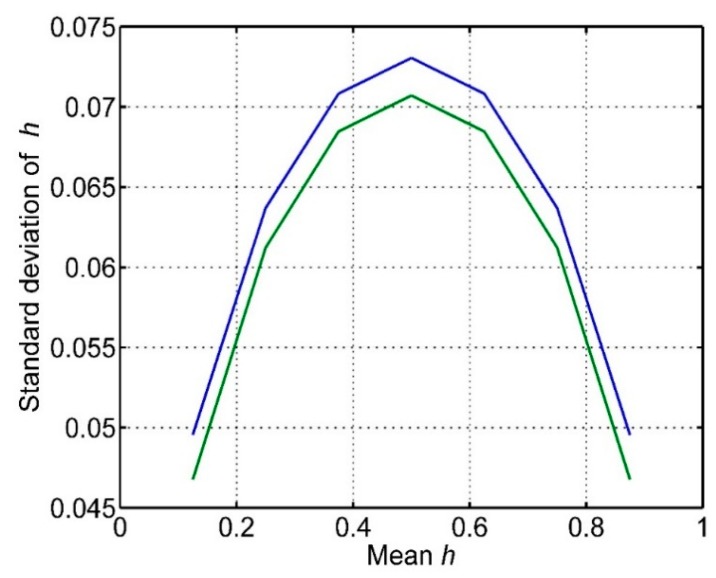
The standard deviation for hit success rate h for the values of $\bar{h}$ plotted in Figure 15 (blue line). Also shown is the theoretical standard deviation from Equation (18) (green line).

It is clear that the variance of *h* varies with $\bar{h}$, being larger for $\bar{h}$ = 0.5 than for smaller or larger $\bar{h}$. The standard deviation of *h* is plotted in Figure 16. This shows that the variability of success in hitting a target is nearly twice as much when the mean value of *h* is 0.5, compared with very small mean *h* or very large mean *h*. The reason is that *p_M_* has a variance proportional to (1 − *h*) and *p_H_* has a variance of proportional to *h* so when either *h* or (1 − *h*) is small, the product *p_M_ p_H_* is narrow. The variance of *h* is given by the simple relation:

(18) $$\sigma_{h}^{2}=\frac{\bar{h}\left(1-\bar{h}\right)}{\lambda S}$$

### Risk: The Uncertainty in Cost

In practice, actuator spacing and platform speed are well known, but there is uncertainly in hit success *h*, as shown in Equation (18). This gives rise to uncertainty in cost function, which is a measure of risk associated with operating the equipment. For the two cost function examples given above:

(19) $$\begin{array}{l} {\left(\rho \gamma \right)}^{u}\left(c-\bar{c}\right)=\left[\left(1-h\right)-\left(1-\bar{h}\right)\right]\\ {\left(\rho \gamma \right)}^{2u}\sigma_{c}^{2}=\sigma_{h}^{2}=\frac{\bar{h}\left(1-\bar{h}\right)}{\lambda S}\\ \frac{\sigma_{c}^{2}}{{\bar{c}}^{2}}=\frac{\bar{h}}{\lambda S\left(1-\bar{h}\right)} \end{array}$$

which shows that the highest relative uncertainty in cost occurs for high hit success rate.

## 5. Conclusions

An analytic model has been developed for the success rate *h* of a robotic actuator on a moving platform hitting randomly spaced targets of uniform circular shape and size. The model results in a single, simple, algebraic equation for *h* in terms of two dimensionless parameters, ρ and γ. These parameters represent the actuator spacing and the distance moved between actuator fire commands, normalized by the target diameter. The equation for *h* completely describes the mean characteristics of this system, providing an easy to use guide for robotic platform design.

If the actuator spacing is less than the target diameter, it is found that 100% success will be achieved if γ^2^ + ρ^2^ ≤ 1. This is not immediately intuitive. If the actuator spacing is greater than the target diameter, then the highest target hit success rate is 1/ρ.

The concept of a cost function is introduced. This function, which should be minimized to minimize real system operating costs, is in general a complicated function of ρ, γ, and *h*. For example, in an agricultural spraying system, the cost of spray units and their depreciation, the time taken to complete a spraying operation, and the percentage of missed targets, as well as over-spraying, all need to be included in the cost function. The cost function is a curve representing constraints, on a plot of success rate *h versus* ρ and γ. For a simple cost function requiring only that the success rate not be lower than a threshold *h*_0_, a simple formula has been developed giving practical limits on actuator spacing and operational speed. While the cost function is an important design tool, it is very difficult to give examples based on actual commercial systems, since the financial details are invariably commercially sensitive.

The analytic model gives a single equation for the *mean* success rate for hitting targets. In practice, since the target locations are random, there will be variation around this predicted success rate for any given actuator spacing and operating speed. A model has been developed which describes this variation and shows that it is described by a simple dependence on the mean number of targets whose centers lie within a unit area: more densely spaced targets reduces the variation in target hit success rate. This uncertainty in success rate propagates through into uncertainty in cost.

## List of Symbols:

*c*	Cost function—a measure of the overall financial cost of targeting
*g*	Probability density function of the hit success rate
*f*	Useful geometric function of dimensionless parameters
*h*	Fractional success rate of hitting a target
*h*_0_	Lower limit of success rate considered acceptable
*h*_min_	Critical value of success rate = 4/5
$\bar{h}$	Mean hit rate
*m*	The number of targets missed
*n*	The number of targets hit
*p*	Probability of a given number of targets within an area
*p_H_*	The probability of a given number of targets hit
*p_M_*	the probability of a given number of targets missed
*r*	Radius of target
*R*	Half spacing of actuators
*S*	Dimensionless area swept out by the actuator beam
*t*	Time
*u*	Power to allow for non-linear dependence of cost on parameters ρ and γ
*v*	Actuator speed
γ	Dimensionless measure of actuator speed
γ_0_	Upper limit of parameter γ giving a hit rate above
γ_0,min_	The lowest value of parameter γ_0_ for which hit success *h*_0_ can be achieved
λ	Mean number of targets whose centers lie within a unit area
θ	Sector angle
ρ	Dimensionless measure of actuator spacing
$\sigma_{c}^{2}$	Variance of cost function
$\sigma_{h}^{2}$	Variance of hit success rate
τ	Time interval between actuator firings
